# Detection of Infertility-related Neutralizing Antibodies with a Cell-free Microfluidic Method

**DOI:** 10.1038/srep16551

**Published:** 2015-11-20

**Authors:** Klaus Eyer, Katharina Root, Pascal E. Verboket, Petra S. Dittrich

**Affiliations:** 1 Department of Chemistry and Applied Biosciences, ETH Zurich Switzerland; 2 Department of Biosystems Science and Engineering, ETH Zurich Switzerland

## Abstract

The unwanted emergence of neutralizing antibodies (nAbs) against an endogenous or a therapeutic protein can result in deficiency diseases or therapy failure. Here, we developed a cell-free microfluidic method for the sensitive detection and quantification of nAbs in human serum that are associated with infertility. We used cell-derived vesicles containing the luteinizing hormone (LH)/choriogonadotropin receptor (LHHCGR) to detect nAbs against LH. The method exploits the entire cellular signal amplification mechanism, and facilitates the detection of as little as 0.44 nM of LH-nAb (*K*_d_ 1.5 nM) in human serum matrix within only 15 minutes. In addition, dose-response curves can be generated in less than 2 hours to evaluate the nAB concentration and dissociation constant. The developed system is devoid of problems associated with cell-based assays and we believe that this simple effect-directed analysis can be used in clinical environments, and is adaptable to other hormones or cytokines and their respective nAbs.

The recent developments of protein therapeutics hold significant promise for improving therapy regimen and reducing side effects^1^. However, the immune system can recognize these peptide agents and respond in an undesired way by inducing the production of antibodies, which is referred to as unwanted immunogenicity^2^,^3^. The emergence of such anti-drug antibodies (ADAs) can impact the pharmacokinetic as well as pharmacodynamic properties of the drug^4^ and can lead to therapy failure^5^,^6^,^7^. In addition to their emergence during therapeutic regimes, antibodies can be also produced against endogenous proteins. The emergence of such antibodies can inhibit the functionality of the protein, e.g. by hindering the binding to its receptor, which can lead to autoimmunity and deficiency diseases^8^,^9^. Depending on their interaction with the protein, ADAs can be divided into binding antibodies (bAbs) and neutralizing antibodies (nAbs). bAbs bind to their target protein without altering the affinity of the target protein to its receptor, whereas nAbs impede the biological effect by effectively inhibiting the formation of the protein-receptor complex. Although many advances have been made in the understanding of the immune system, as well as the production and quality control of biopharmaceuticals to reduce the risk of immunogenicity^10^, the problem of immunogenicity and nAb generation remains challenging^2^.

The efficient detection of ADAs would allow to assure therapy success and to diagnose deficiency diseases in an early state. Such a detection system holds promise to efficiently monitor drug therapy, and could massively decrease costs in health care. Currently, the presence of nAbs can be detected either by symptomatically monitoring or by the examination of the patient’s serum^3^. Gupta *et al.*^4^ recommended a two-step serum testing and characterization strategy for the detection of ADAs, and discrimination of bABs and nAbs. The first test series comprises screening and confirmatory immunoassays^11^,^12^, capable of detecting ADAs that can bind to the protein. These cell-free methods can be performed at the point-of-care, and are fairly reliable and robust^13^,^14^. In addition, other formats, such as electrochemiluminescence assays can be used for the detection at this stage^15^,^16^. Despite all the advantages of immunoassays and similar formats, the drawback of only testing the binding of the agent has to be mentioned. As a consequence, these methods are often less sensitive in the absence of signal amplification and lack the testing of the actual biological function. Furthermore, these first tests are normally only capable to detect ADAs, but not to discriminate between bAbs and nAbs. After initial screening for ADAs, the second stage comprises cell-based formats to discriminate between bAbs and nAbs. Cell-based systems are more sensitive because of the efficient conversion of the binding event into a signalling event, i.e. one binding event leads to the generation or influx of many signalling molecules. A number of cell-based assays have been reported that identify and characterize nAbs, such as cell-surface interactions that can be measured via fluorescence activated cell sorting (FACS)^16^, the conversion of second messengers^17^, cell proliferation, differentiation and apoptosis assays^18^,^19^, expression of cellular markers or enzymes^20^,^21^, or the release of cytokines^11^ among others. Although very sensitive, cell-based assays are more prone to interfering processes from sample matrix. To circumvent this problem, strategies to eliminate the disturbing factors by sample pre-treatment have been developed^22^,^23^. These strategies enable the detection of nAbs even in the presence of high antigen levels in serum. However, pre-treatment steps increase the length and complexity of the protocols, and can be a source for errors. Further disadvantages of cell-based assays are their costs, their equipment needs and the difficulty of standardization.

To overcome the drawbacks, we introduce a new analytical method for the detection of nAbs. Our protocol is as simple as an immunoassay, but provides the sensitivity of cellular assays. We use cell-derived vesicles produced from adherent cells via treatment with cytochalasin b, where membrane proteins and cytosolic proteins are preserved within the cell-derived vesicles^24^,^25^. These vesicles are immobilized inside a microfluidic chip (Fig. 1A), allowing precise control of supplied fluids due to the microfluidic environment. The cells are genetically modified so that a strong bioluminescent signal is generated in response to a stimulus, and also in the vesicles afterwards. The cell-derived vesicles can be produced in large batches and stored at least for two months^26^, i.e. no permanent cell culture is needed and laboratory and user variation can be reduced by the batch-wise production. Furthermore, influence of the sample matrix on the signal is reduced because many interfering processes are no longer present. This reduction includes processes where other cell compartments are involved as well as processes, where matrix molecules bind to other, low abundant receptors and induce signalling. Compared to the high abundant target receptor, these low abundant receptors are only present on a small fraction of the cell-derived vesicles and the influence of their activation is minimized due to the compartimentalization.

We employ the device to detect nAbs against the luteinizing hormone (LH). LH is a protein hormone of the gonadotropin family. Antibodies against LH are regularly found in female sera, and the emergence of these antibodies can lead to infertility^27^ as well as fertility-treatment resistance^28^. Shatavi *et al.*^29^ determined the prevalence of gonadotropin autoantibodies in formerly inexplicable infertility cases, and they found a significantly higher prevalence of auto-LH and FSH antibodies in the infertile population (60% vs. 15%), and an even higher prevalence (67%) in women receiving fertility treatment. Therefore the presence of such antibodies may represent a marker of autoimmunity associated with infertility. Additionally, our method can distinguish between bAbs and nAbs, and therefore can be used to further validate such a marker.

## Results and Discussion

### Characterization of the cell-derived vesicles

**Figure 1:**
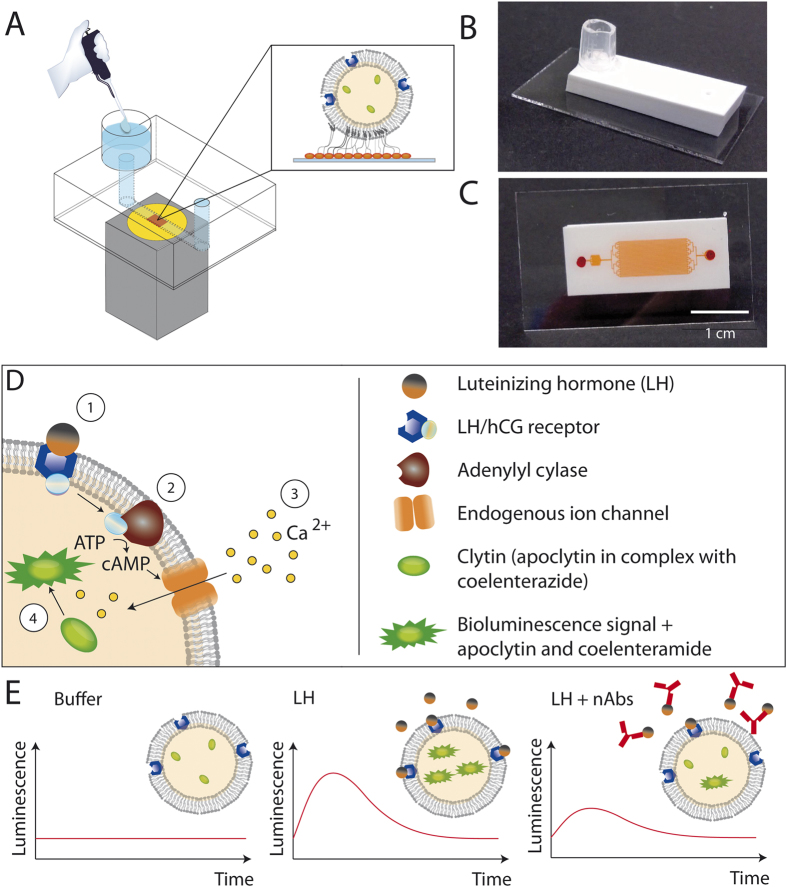
Detection of nAbs. (**A**) A microfluidic device with one reservoir for sample introduction and one outlet channel is directly mounted onto a photomultiplier tube so that the channel is aligned to the photosensitive area (yellow). Cell-derived vesicles are immobilized via cholesterol moieties onto the surface (red square) (not to scale). (**B**) Picture of the microfluidic device that is made of a PDMS-TiO_2_ mixture. (**C**) View of the microfluidic device from the bottom side. For illustration, the channels are filled with red food dye. The channel system consists of an inlet, a microfluidic filter, the sensing area (16 parallel channels) and an outlet. The sensing area is optimized to maximize the surface area to immobilize vesicles. (**D**) Four-step biochemical reaction pathway. The cell-derived vesicles contain the receptor for LH (LHHCGR), adenylyl cyclases and endogenous ion channels as well as intravesicular clytin as Ca^2+^ sensor. Upon binding of luteinizing hormone to the receptor (1), the G-protein coupled receptor is activated and adenylyl cyclases produce cAMP (2) that in turn activates endogenous ion channels to allow calcium influx (3). The increase in intravesicular calcium concentration activates clytin to generate bioluminescence by the conversion of coelentrazine to coelenteramide (4). (**E**) Schemes of the luminescence signal over time in the presence of buffer, LH and LH plus nAbs.

The employed method for the production of cell-derived vesicles resulted in structures that contain membrane receptors as well as intracellular components^25^,^26^. The cell-derived vesicles were typically 600–900 nm in diameter, as confirmed by dynamic light scattering (see SI Table 1). This size distribution has been confirmed by different studies^24^,^25^,^26^. In the following, we describe several control experiments to verify the presence of all essential components for the assay presented here: (1) the LHHCG receptor needed for binding of LH, (2) the adenylyl cyclase (AC) for the generation of cAMP, (3) endogenous ion channels for calcium influx and (4) clytin for the generation of light (see also Fig. 1).

**Figure 2:**
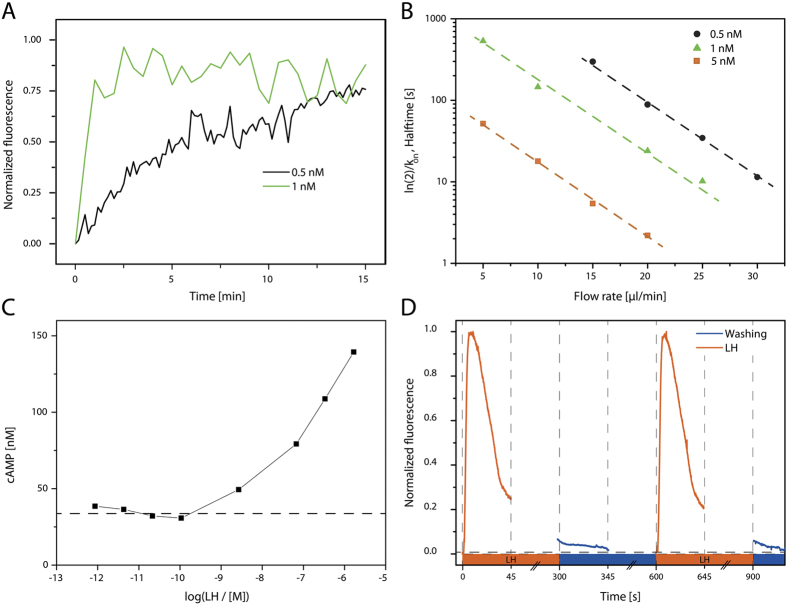
Characterization of the cell-derived vesicles. (**A**) Cell-derived vesicles were immobilized on-chip and fluorescently labelled luteinizing hormone was supplied. The increase of fluorescence over time indicates that the LHHCGR receptors are present and bind the hormone (flow rate: 20 μl/min). (**B**) Binding kinetics at the vesicular membrane in dependence of the hormone concentration and the flow rate. (**C**) Formation of cAMP, determined with the cAMP Glo™ assay. The increase in cAMP proves that the receptor for luteinizing hormone and the adenylyl cyclase is functional (n = 2). (**D**) Determination of Calcium influx, determined with the Fluo-4 assay. The influx is reproducible, even when the vesicles are used for several runs (LH addition in the reservoir of the chip at t = 0 and 600 s).

First, the presence and functionality of the LHHCG receptor was confirmed by visualizing the binding of the hormone to the receptor. To visualize binding, we labelled LH with lissamine rhodamine B, and measured the accumulation of fluorescence on immobilized cell-derived vesicles. The vesicles were immobilized on the surface in a characteristic pattern using micro-contact printing, which allowed for the correction of non-specific accumulation on the surface^30^. The binding of the hormone was monitored (Fig. 2A) and the increase in fluorescence fitted exponentially to extract the association rate of this process, *k*_on_. As visible in Fig. 2B, the association rate was depending on the flow rate as well as on the concentration of the hormone, i.e. the total mass transfer towards the immobilized cell-derived vesicles. Firstly, this finding showed that the hormone-receptor interaction was not significantly altered in the cell-derived vesicles. Secondly, the accumulation of the hormone on the vesicular membrane was solely depending on the concentration thereof (when constant flow rate is applied), so that the extraction of quantitative information was possible. Lastly, a control experiment using vesicles derived from HEK293 cells (LHHCGR negative) showed no significant accumulation of the hormone, and no accumulation was found on a surface without immobilized vesicles (see SI Fig. 1).

The next step in the signalling cascade was the formation of the second messenger cAMP after receptor binding. We used a commercial detection kit for cAMP in a plate reader format (see Materials and Methods for more information). In these experiments, the cell-derived vesicles were suspended in HBSS and the hormone was added in different concentrations. First experiments revealed only very small amounts of formed cAMP, with a large variation from experiment to experiment. We assumed this fact to be due to variations in the intravesicular ATP concentrations. Due to the absence of efficient metabolism in the derived vesicles and therefore constant ATP concentrations, the amount of produced cAMP depended on the time between production and actual use in the experiments. When adding external ATP (1 mM), the formed cAMP amounts as well as the reproducibility of the assay were greatly increased; indicating that ATP was able to cross the vesicular membrane, which was in accordance with our findings in former studies (see also SI Fig. 2)^26^. In the final experimental series, the cell-derived vesicles were stimulated in the presence of externally added ATP and a dose-response curve was determined for the addition of the hormone (Fig. 2C). Without luteinizing hormone, a basal cAMP concentration of around 35 to 40 nM could be found. We assumed that the partial inhibition of phosphodiesterases due the addition of IBMX (3-isobutyl-1-methylxanthine) resulted in this constant level, as well as an amount of the externally non-converted ATP increased basal light output. In conclusion, we could show that cAMP was formed inside the cell-derived vesicles as one essential intermediate in the reaction pathway.

The next step in the cascade towards intravesicular bioluminescence was the influx of calcium ions due to the opening of endogenous ion channels, which in turn activated the enzyme clytin. To confirm the influx of calcium ions, cell-derived vesicles were loaded with Fluo-4 AM. After de-esterification, the formed Fluo-4 moiety is trapped inside the vesicles and acts as a calcium-concentration sensitive fluorescent dye. The loaded vesicles were immobilized inside the microfluidic channel, and fluorescence was measured over time. As visible in Fig. 2D, intravesicular fluorescence was increasing upon the addition of LH. Furthermore, we found that the increase in fluorescence signal was transient and returned to base level when LH was removed from the vesicles by supply of LH-free buffer. Lastly, we characterized whether the vesicles can be repeatedly stimulated. This feature would allow using the same immobilized vesicles on the same chip for multiple test and calibration solutions, therefore increasing reproducibility and decreasing workload. Indeed, repeated experiments proved that the calcium influx and efflux were reproducible (Fig. 2D). The deviations for at least three subsequent experiments were minor, showing that the same immobilized vesicles could be stimulated multiple times.

### Confirmation of reactivity and optimization of the microfluidic detection system

In a series of experiments we confirmed the reactivity of the cells towards the hormone and the neutralizing effect of the commercially bought antibody towards LH. Detailed information can be found in the supporting information (SI and SI Fig. 4).

Due to the small size of the cell-derived vesicles and low volumes in microfluidics, bioluminescence outputs tend to be lower as compared to stimulation within well plates using cells. To allow for sensitive detection, the microfluidic platform and the detection setup had to be optimized. For fabrication of the microfluidic platform, we used a highly reflective TiO_2_—PDMS composite, which we developed and characterized recently^31^ (See Fig. 1B). The microfluidic chip comprises sixteen 100 μm wide channels in parallel (Fig. 1C) to maximize the number of cell-derived vesicles that can be immobilized on one chip and thereby maximizing the signal that can be obtained per chip. Furthermore, we developed a small, portable system containing a photomultiplier tube (PMT) placed in a light-tight enclosure to reduce background signal (SI Fig. 4). Besides the detector, no further optical components are required, resulting in a straightforward and small setup.

### Bioluminescence measurements on the microfluidic platform

**Figure 3:**
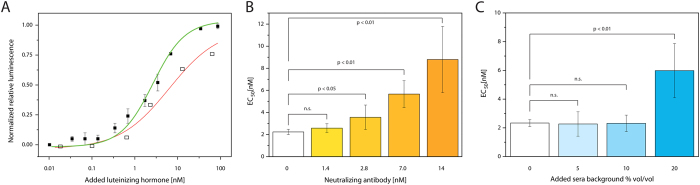
Addition of LH, nAb and human serum to immobilized cell-derived vesicles. (**A**) Dose-response curve of cell-derived vesicles for different LH concentrations, with an EC_50_ value of 2.11 ± 0.24 nM (black squares, n = 3, fitted curve green). For comparison, the dose response curve of cells is shown as well (fit shown in red, n = 2, shown in hollow rectangles, for the full curve please refer to SI Fig. 1), with an EC_50_ value of 10.2 ± 2.4 nM. (**B**) EC_50_ values for different concentrations of nAb added to the mixtures. The addition of antibody resulted in a shift of the dose-response curves (as shown in A) towards larger LH concentrations, i.e., larger EC_50_ values. The presence of 2.8 nM neutralizing antibody is enough to have a significant change in response (n = 3 for 0 nM, n = 1 for others). (**C**) EC_50_ values for different concentrations of human serum matrix added to the mixtures. Up to a serum concentration of 10% is tolerated without changing the respective dose-response curves. At a serum concentration of 20% vol/vol, the fitted EC_50_ value deviated significantly from the value measured in buffered solution (n = 3 for 0% vol/vol, n = 1 for others).

After confirmation of the vesicles’ functionality and optimization of the setup, the method was validated for the detection of LH by means of a bioluminescence assay as sketched in Fig. 1C. First, measurements with a constant LH concentration were performed repeatedly on the same chip, with intermediate washing steps to ensure the signal was constant over time (see SI Fig. 5). Next, the dependence of the bioluminescent signal was measured in the presence of different LH concentrations. A complete dose-response curve was obtained on one single chip using the same immobilized vesicles repeatedly. These measurements were repeated on three different chips and with three different batches of cell-derived vesicles, and the results are shown in Fig. 3A. Fitting of the data revealed a half effective concentration (EC_50_) of 2.43 ± 0.20 nM. This EC_50_ value is about 5 times lower than the one found in the cell experiments before (shown in red, EC_50_ 10.2 ± 2.4 nM). The deviation can be explained by accumulation of the hormone at the vesicular surface under flow conditions (vesicle experiments), whereas the cell experiments were performed under stationary conditions. In other words, in the stationary cell experiments the concentration of free LH decreased over time ([LH]_eq_ = [LH]_initial_ − [LH]_bound_), whereas in the microfluidic device, the free LH concentration is constant due to the continuous supply of LH, and therefore, more LH bound to the receptors, further increasing the sensitivity of our developed method.

Next, dose-response curves were measured in the presence of nAbs. The addition of a neutralizing agent decreased the concentration of free LH that could interact with the receptor and hence, resulted in a shift of the curves towards higher concentrations. The shift can be explained since higher amounts of LH were needed to achieve the same stimulation due to the partial neutralisation. Indeed, higher EC_50_ values were derived from the curves after fitting, increasing when the concentration of nAbs was raised (Fig. 3B).

### Detection of nAbs in serum

The final aim of the method was the detection of nAbs in patient serum. Therefore, we used pooled female serum and measured the LH dose response in presence of serum matrix. Variations of serum dilutions were tested with the goal to use as high amounts of serum matrix as possible. The fitted EC_50_ values of the respective dose-response curves are shown in Fig. 3C. In general, the data showed more noise upon serum addition (visible in the higher standard deviations), which we expected by using this more complex sample matrix. Proteins and other molecules present in the serum can interact with their receptors in the vesicular membrane, and influence the assay outcome. However, due to compartmentalization of the assay into small vesicles the matrix influence was not significant. No alteration in the EC_50_ value could be detected up to a 10% serum concentration when compared to the pure buffer.

**Figure 4:**
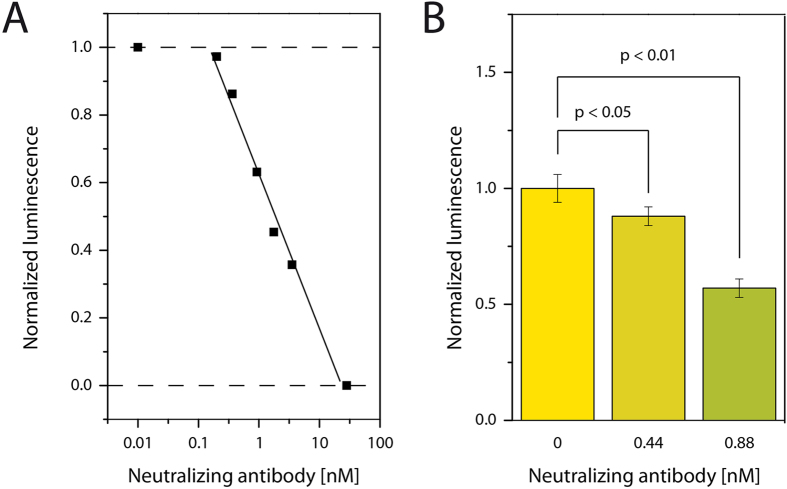
Detection of neutralizing antibody in human serum. (**A**) Different concentrations of nAb were added to a constant concentration of LH (1.78 nM) in 10% human serum background (n = 1). As visible, signal decreased upon increase of nAB concentration. (**B**) Repeated experiments using different chips, different batches of cell-derived vesicles and chemicals (n = 4) proved the reliable detection of 0.44 and 0.88 nM nAb in the presence of 10% serum matrix.

For determination of the limit of detection (LOD), we conducted experiments using a constant concentration of LH (50 ng/ml), and added different amounts of nAbs to the solution. The decrease in the bioluminescence signal with increasing nAb concentration was depicted in Fig. 4A. As visible, the addition of ≥28 nM antibody resulted in a luminescent output equal to the one without LH. Together with the dissociation constant of the antibody, which we obtained by SPR measurements (1.53 ± 0.67 nM), we could calculate that the concentration of free LH was indeed only 13 pM and hence, too low to be detected (see also Fig. 3A). On the other hand, the addition of ≤10 pM nAb resulted in a bioluminescence output that was equal to the one without nAb (due to the very low neutralization capacity of the antibody). Here, the neutralized proportion of LH was too small to influence the measurements. Fast qualitative detection of nAbs is therefore possible by simply comparing the bioluminescence signal of the diluted patient serum with nAbs-free diluted pooled serum at a given LH concentration.

For confirmation of the LOD in the range of a few hundred pM nAb and show the reliability of the method, we chose two low concentrations of antibody (0.44 and 0.88 nM) and compared the results with blank samples. The experiments were repeated 4 times on different microfluidic devices with different batches of cell-derived vesicles of different cell passages. As visible in Fig. 4B, the commercially bought nAb could be reliably detected at a concentration of as little as 0.44 nM. The LOD was calculated using two different methods,(i) the unpaired t-test, which resulted in the LOD of 0.44 nM based on our experimental results in Fig. 4B, and (ii) by the method proposed by Armbruster *et al.* (ref. 32), which results in a LOD of 0.29 nM. Giving the dilution of the serum, the needed initial concentration for detection of this antibody in human serum is 10 times higher, i.e. in the low nM range. Hence, our method has an excellent sensitivity. Previous cell-free methods without amplification developed for other nAbs typically allowed detection in the micromolar to higher nanomolar range, whereas multistep cell-free methods with signal amplification such as ELISA can be reliably performed down to the lower nanomolar range^13^,^14^,^15^. Cell-based methods are usually reliable in the larger nM range (typical range between 3 μM and 3 nM^17^,^18^,^19^,^20^). In contrast, our microfluidic method with cell-derived vesicles facilitates, in one step, the detection of antibodies in the lower nM range and their classification as neutralizing antibodies.

**Figure 5:**
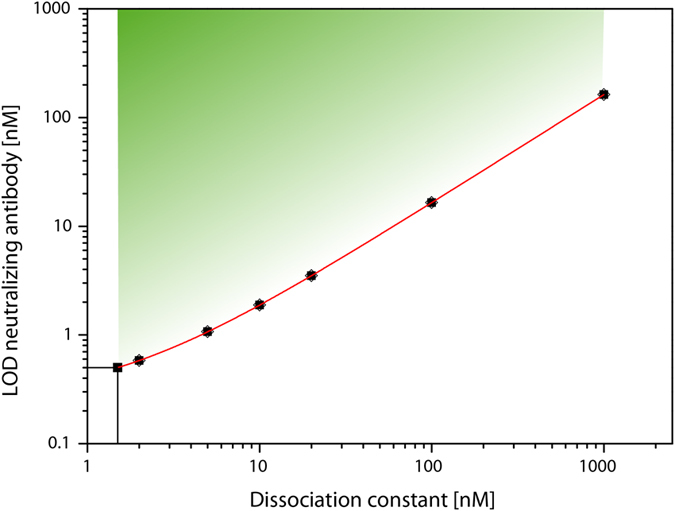
Limit of detection for nAbs with different binding strengths. For the calculations, we used the *K*_d_ of our tested antibody and calculated the amount of free luteinizing hormone (assumption 1:1 binding). For all of the other values, this free concentration was fixed and the other parameters (concentrations of complex and free antibody) were varied until the calculated *K*_d_ was equal to given *K*_d_ values. The fitted red line indicates the limit of detection at a given dissociation constant.

Despite the dilution of the serum, the limit of detection is clearly below the concentration of antibodies that can be typically found in human serum (around 10 and 400 nM for a certain antibody species^33^). However, in clinical samples, the dissociation constant of the antibody can vary significantly, which will influence the limit of detection accordingly. Figure 5 shows the relation of the nAb dissociation constant and the limit of detection for our method (see SI for the calculation). A wide range of nAbs can be detected, both concentration and affinity wise, which are in accordance with the respective values from former studies^34^,^35^,^36^. To conclude, our cell-free method has the sensitivity that is required to detect clinical relevant nAbs.

### Estimation of the concentration and dissociation constant of an unknown nAb

Besides the detection of the *presence* of nAbs, the concentration of a nAb with unknown K_d_ can be determined with this method using the procedure as follows. The bioluminescent signal depend on the concentrations of LH, nAbs and the dissociation constant (*K*_d_) of the complex. When several dose-response curves are recorded each at different concentrations of LH, the dissociation constant as well as the concentration of total nAb can be estimated by using an equation solver and minimizing the square residues. This is exemplarily shown in the Supplementary Information for the antibody that was available in our study. Here, the whole protocol required about 2 hours, and provided results in good agreement with the expected values.

## Conclusion and Outlook

We successfully developed a cell-free method for the detection of Abs that combines the sensitivity of cell-based methods with the ease of handling of cell-free methods. The cell-derived vesicles can be produced in larger batches and stored for longer time periods (up to 8 weeks tested), hence providing an attractive and less laborious alternative to current cell-based assays. In our method, continuous cell culturing and medium supply or heating during measurements is not required, and there are no disturbing influences on the results originating from cell division and growth or harmful effects of the tested compounds. With our developed analytical platform, we achieved a detection limit of as little as 0.44 nM of neutralizing antibody (with the K_d_ of 1.5 nM) in a 10% serum matrix in only 15 minutes. Furthermore, testing different patient’s sera on the same microfluidic chip is possible, therefore optimizing the throughput in clinical settings. For a more thorough characterization of the patients’ nAbs, dose-response curves can be generated on the same microfluidic device within two hours, and the dissociation constant as well as the concentration of the nAb can be estimated. We are confident that the method will prove successful for clinical samples.

Our method shows the first use of cell-derived vesicles in a diagnostic context, and overcomes limitation of cell-based assays. It is as sensitive as a cell assay since the entire cellular signal amplification pathway is exploited after its activation through the binding of the hormone to the G-protein coupled receptor (GPCR). We believe that this cell-free analytical method can be applied for the detection of other (non-LH) nAbs, provided that the binding of the drug/protein that is neutralized activates a calcium pathway to trigger the bioluminescence generation. At the same time, it can be used to study the binding of compounds to receptors e.g. for effect-directed analysis of environmental sample, or in the drug discovery process (e.g binding of drug candidates to GPCR).

## Materials and Methods

### Reagents

SU8 and developer were obtained from Microchem (Newton, MA, USA). Poly(dimethylsiloxane) (PDMS, Sylgard 184 elastomer kit) was sourced from Dow Corning. 1*H*,1*H*,2*H*,2*H*-perfluorodecyl-trichlorosilane and adenosine were procured from ABCR (Karlsruhe, Germany). DMEM/F12, fetal bovine serum (FBS), geneticin sulfate (G418), N-[4-[6-[(acetyloxy)methoxy]-2,7-difluoro-3-oxo-3H-xanthen-9-yl]-2-[2-[2-[bis[2-[(acetyloxy)methoxy]-2-oxoethyl]amino]-5-methylphenoxy]ethoxy]phenyl]-N-[2-[(acetyloxy)methoxy]-2-oxoethyl]-,(acetyloxy)methyl ester (Fluo-4 AM), lissamine rhodamine b sulfonyl chloride, 100x non-essential amino acids, RPMI and 0.05% trypsin-EDTA were obtained from Invitrogen. Human serum pooled, female, pre-menopausal (product number SF-123-H, batch number M410239) was obtained from Sera laboratories International (West Sussex, UK). Adenosine 3′,5′-cyclic monophosphate (cAMP), adenosine-5′-triphosphate disodium salt hydrate (ATP), biotinylated and bovine serum albumin (bBSA and BSA), coelenterazine hcp, cytochalasin b, dimethlyformamid (DMF), 3-isobutyl-1-methylxanthine (IBMX) and dipyridamole were obtained from Sigma Aldrich. Titanium dioxide powdered was obtained from Fluka AG. Hygromycin and puromycin were purchased from Merck Millipore. Luteinizing hormone (LH) was a gift from Merck Serono (Italy). LHB monoclonal antibody clone L^+^ was obtained from Abnova (Taipei, Taiwan). Avidin was purchased from AppliChem (Darmstadt, Germany). Biotin-PEG-cholesterol (M_w_ 3400 Da) was obtained from Nanocs (New York, NY).

### Cell culture

The cell line HEK293 HTS233L (ChemiBrite™ LH glycoprotein hormone receptor stable expressing cell line, Millipore, USA) was used for experiments. The cells were sub-cultivated twice a week in a ratio 1:5, using 0.05% trypsin-EDTA for detachment during the process. The used culture media was DMEM/F12 with added 10% fetal bovine serum, 1x non-essential amino acids, 1 μg/ml puromycin, 200 μg/ml geneticin and 100 μg/ml hygromycin.

### Cell stimulation experiments

For cell stimulation experiments, the cells were seeded in μslides (ibidi, München, Germany) 24 hours prior to stimulation. Before stimulation, the cell media was removed, the cells were washed with HBSS once and afterwards incubated at 37 °C for 1 hour with 1 μM coelentrazine hcp in HBSS. Afterwards, the indicated amount of luteinizing hormone was added to the cell layer and luminescence was recorded using an EMCCD camera (887, Andor, Belfast) at an exposure time 0.95 s (gain 200) on an inverted microscope (IX70, Olympus) for 2 minutes. Signal was corrected for the light intensity measured before adding the hormone.

### Cell-derived vesicle formation

For the production of cell-derived vesicles, the supernatant was aspired and the cell layer was washed with serum free RPMI. Afterwards, the cells were incubated in the presence of 10 μg/ml cytochalasin b in RPMI for 20 minutes. The flask was then gently tapped to release the vesicles into suspension, and the supernatant was collected and filtered through a 10 μm filter (CellTrics, Partec, Germany). Afterwards, the vesicles were introduced to the microfluidic chip (see section surface modification).

### Master fabrication

SU-8 2015 was processed to a height of 20 μm and, after exposure to UV light (150 mJ/cm^2^, measured at 365 nm) in a mask aligner (MA-6 mask aligner, Karl Suess), developed using SU-8 developer. Master forms were silanised by storing the wafers overnight in an desiccator with 50 μl of 1*H*,1*H*,2*H*,2*H*-perfluorodecyl-trichlorosilane at 100 mbar.

### Chip fabrication

Microfluidic devices were prepared using the Sylgard 184 elastomer kit. The two components were mixed in a ratio of 10:1, and 15.4% w/w TiO_2_ was suspended inside the mixture^31^. The suspension was thoroughly mixed, degassed for half an hour and subsequently poured onto the master mold. The assembly was put at 80 °C for 2 hours to cure. After curing, the elastomer was peeled from the wafer and cut into the respective devices. Access holes for fluidic connections were punched with a biopsy puncher (1.5 mm diameter, Miltex, York, PA, USA), and a reservoir was attached by using semi-cured PDMS. The device was exposed to 80 °C for another hour. To close the channels, the PDMS part was bonded to a glass slide after plasma activation for 45 seconds at 18 W and 0.75 mbar (Harrick Plasma Cleaner PDC-32G, Ithaca, NY, USA). The final assembly was put at 100 °C for 10 minutes to assure a stable bond.

### Surface modification

To immobilize cell-derived vesicles, the surface was modified according to Kuhn *et al.*^37^. Here, biotinylated BSA (0.5% w/w) was introduced into the chip by centrifugation (800 g, 5 min, 30 min incubation), followed by a second centrifugation step with BSA (4% w/w) to inactivate the remaining free surface (800 g, 5 min, 30 min incubation). During the incubation steps, biotinylated BSA and BSA adsorb spontaneously to the glass and PDMS. Next, the chip was attached to a syringe pump (neMESYS, Cetoni, Germany) using custom-made metal connectors and PTFE tubing. Then, 1 μM avidin was added to the reservoir and drawn through the device with a flow rate of 5 μl/min for 20 minutes. Afterwards, the remaining solution inside the reservoir was exchanged to buffer containing 7 μM biotin-PEG-cholesterol and drawn through the chip (5 μl/min, 20 minutes). Afterwards, the device was washed with buffer. After the washing step, the vesicle suspension was introduced into the device (2.5 μl/min, 20 minutes). The vesicles are captured by the cholesterol linker that partitions into the vesicle membrane. We assume that the surface is fully covered after 20 minutes. After a final washing step with buffer the device is ready for experiments.

### Fluorescent labelling of LH

For labelling with lissamine rhodamine b, the hormone was dissolved in carbonate buffer pH 9.0 at a concentration of 940 μg/ml. Freshly dissolved lissamine rhodamine b sulfonyl chloride in water-free DMF was added in a molar excess of 20, and was incubated in a darkened lab at room temperature while gently shaking for 1 hour. Afterwards, the labelled hormone was separated from the free dye by size exclusion centrifugation (Nanosep 10K, Pall Life Sciences, Ann Arbor, MI). Concentration of the hormone and degree of labelling (DOL) were determined by UV absorption measurements (LH molar extinction coefficient_280 nm_ 12430 (Mcm)^−1^). Degree of labelling was usually between 0.2 and 0.5, and the concentration of the purified labelled luteinizing hormone was around 150 μg/ml.

### Binding assay

For generating parallel streams of different solutions, the solutions were flushed through a chip comprising two inlet channels (width 300 μm, height 20 μm), a measurement channel (width 300 μm) and one outlet. Syringes were filled with HBSS or the respective concentration of fluorescently labelled LH in HBSS. To monitor the binding kinetics of the hormone to its receptor, the total flow rate was varied from 20 to 30 μl/min, but also ratios of flow rates of the two solutions were varied from 5 to 30 μl/min, similar to flow schemes in our former studies to expose the labelled LH subsequently to defined regions inside the channel^30^,^38^. In this way, four independent measurements can be performed successively on each chip. The microfluidic device was mounted on a TIRF microscope (Leica, DMI6000B, Leica Microsystems). Binding of the fluorescent hormone was monitored using the 561 nm laser line (250 ms exposure time, 120 gain, 12% laser power). Fluorescent micrographs were taken every 10 seconds. Data was treated as explained in reference^30^.

### cAMP measurements

For the measurement of produced cAMP inside the cell-derived vesicles, a commercially available cAMP assay kit was used (cAMP-Glo™ assay, Promega). For this experiment, c ell-derived vesicles were freshly prepared, and 10 μl of the resulting vesicular solution was added to a 96-well plate (flat bottom, black, Corning). To stimulate cAMP responses, the vesicles were incubated with the indicated amount of hormone for 15 minutes. Phosphodiesterases were inhibited by adding 100 mM IBMX to the incubation buffer. Afterwards, the plate was analysed as described in the kits manual (i.e. the vesicles were lysed). To measure the generated luminescence in this assay, the plate was read using a Synergy HT plate reader (Bio-TeK, Winooski). A calibration curve was performed simultaneously to convert measured luminescence intensities into cAMP concentrations directly.

### Calcium measurements using Fluo-4

To visualize calcium influx into the cell-derived vesicles, freshly prepared vesicles were incubated with 1 μM Fluo-4 AM. Afterwards, the vesicles were immobilized on-chip as described above. The microfluidic device was mounted on an inverted microscope (IX70, Olympus, Switzerland), fluids were pipetted into the reservoir and drawn trough the chip (10 μl/min) for 5 minutes. For measurements, a concentration of 940 ng/ml (33.6 nM) of luteinizing hormone was added to the cell-derived vesicles. Fluorescence (Ex: 455/70, Dichro 494 LP, Em: 515 LP) was measured for 1 minute (8 frames per second, exposure time 100 ms, gain 150). Afterwards, the chip was flushed with HBSS buffer without hormone for 5 minutes (10 μl/min). The procedure of activation and washing was repeated four times. Bleaching curves were obtained at the end of the series, however, the bleaching was found to be negligible for the experiments. Data was analysed using ImageJ, and processed in Microsoft Excel.

### SPR measurements

Determination of the dissociation constant of the used neutralizing antibody (LHB monoclonal antibody clone L^+^) was made using surface plasmon resonance (SPR) measurements (Biacore Life Sciences, GE Healthcare). Here, the antibody was immobilized on the chip surface (Sensor chip CM3), the different concentrations of luteinizing hormone were added and on/off kinetics were monitored over time and fitted with a monoexponential function. From these measurements, the dissociation constant was found to be 1.53 ± 0.67 nM.

### On-chip Bioluminescence measurements using cell-derived vesicles

In all of the mentioned experiments below, the cell-derived vesicles were freshly prepared and immobilized on-chip as described above, and all respective fluids were added to the reservoir and drawn trough the chip at 10 μl/min. Luminescent signals were recorded using a custom-build detection system consisting of a photosensor modul H10722–210 (Hamamatsu, Switzerland) which was implemented into a closed container (see SI Fig. 2). To maximize signal, the microchip was directly placed above the active sensing surface of the photomultiplier tube (PMT). A custom LabView script (National Instruments, Austin, TX) was used for data acquisition. The luminescence signal was recorded for 5 min every 500 ms at a control voltage of 750 mV. The signal from the first three minutes was integrated over time and is referred hereafter as area under the curve (AUC).

If not mentioned otherwise, the incubation buffer always contained 1 μM coelenterazine hcp and 2 mM ATP, whereas ATP was added shortly before measurements to prevent any deviation due to hydrolysis by serum ATPases. After applying a concentration of luteinizing hormone, the immobilized vesicles were washed for 5 minutes using HBSS containing 2 mM ATP (washing buffer). Experimental series under different conditions were performed as described below.

### a) ATP series

To investigate the influence of externally added ATP, different concentrations of ATP were supplied to the vesicles. Here, the LH concentration was constant (940 ng/ml), while ATP concentrations varied. AUCs were normalized to the AUC without any ATP added (see Equation 1). Here, washing buffer contained no ATP.


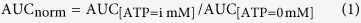


### b) LH addition–dose response

To generate a dose-response curve, different concentrations of luteinizing hormone ranging from 0.01 to 100 nM were added to the immobilized cell-derived vesicles. The recorded AUCs were normalized to their recorded maximal signal (plateau at [LH] = 100 nM, AUC_LH = 100nM_) and minimal signal (basal level at [LH] = 0 nM, AUC_LH = 0_ nM) (see Equation 2).


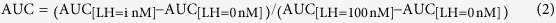


The resulting curve was fitted with a dose-response equation after Hill (see Equation 3),


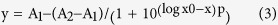


where A_1_ and A_2_ depict the basal and plateau level (here 1 and 0), logx0 the point of inflection and p the steepness of the curve. The point of inflection was further used to calculate the concentration for half-maximal activation (EC_50_).

### c) Sera addition–dose response

Different concentrations of sera (0, 5, 10 and 20% vol/vol) were added to the HBSS buffer to investigate matrix effects. As test serum, pooled pre-menopausal human serum was used. Dilution series of luteinizing hormone concentrations (0.01 to 100 nM) in serum-containing HBSS were supplied to the immobilized cell-derived vesicles. Integrated AUCs were normalized to maximal and minimal signals (Equation 2), and fitted with a dose-response equation (Equation 3). In this series, minimal and maximal signals were measured in serum background.

### d) nAb addition–dose response

Different concentrations of neutralizing antibody (from 0 to 14 nM) are added to various concentrations of LH (from 0.01 to 100 nM). Before using the antibody stock solution, sodium azide was removed from the antibody solution by ultrafiltration (cut-off filter 10 kDa, Nanosep, Pall Corporation, Switzerland), the reason being that the conservator is known to interfere with many cellular processes. Neutralizing antibody and LH solution were mixed and incubated for 15 minutes at room temperature to allow the antibody to bind to its target. Again, integrated AUCs were normalized to the maximal and minimal signals (Equation 2), and fitted with a dose-response equation (Equation 3). Here, the maximal signal was measured in the absence of antibody, and minimal signal in the absence of LH.

### e) Combinatorial approach–dose response

To generate dose-response curves in the presence of serum and different amounts of antibodies, dilution series of luteinizing hormone were made in serum-containing HBSS (constant, 10% sera background, LH concentrations ranging from 0.01 nM to 100 nM), and mixed with various concentrations of nAbs (from 0 to 14 nM). The solutions were incubated for 15 minutes at room temperature, then pipetted into the reservoir and drawn over the immobilized cell-derived vesicles. Data acquisition and analysis was done as before. Here, the minimal signal and the maximal signal without addition of antibody were taken in serum background.

### f) LOD measurements with constant LH

To determine the limit of detection in the presence of sera, luteinizing hormone (50 ng/ml, 1.78 nM) was added to serum-containing HBSS (constant, 10% sera background) in the presence of nAbs (concentration varies from 0 to 44 nM). The solutions were incubated for 15 minutes at room temperature before measurement, and added to the reservoirs and drawn over the vesicles. Data acquisition and analysis was done as before.

## Additional Information

**How to cite this article**: Eyer, K. *et al.* Detection of Infertility-related Neutralizing Antibodies with a Cell-free Microfluidic Method. *Sci. Rep.* **5**, 16551; doi: 10.1038/srep16551 (2015).

## Supplementary Material

Supplementary Information
