# Supplementary material for: Detection of Infertility-related Neutralizing Antibodies with a Cell-free Microfluidic Method
Source: Sci Rep. 2015 Nov 20;5:16551. doi: 10.1038/srep16551 (PMC4653734; doi:10.1038/srep16551)
Supplement: Supplementary Information [file srep16551-s1.pdf]

SUPPORTING INFORMATION

DETECTION OF INFERTILITY-RELATED NEUTRALIZING ANTIBODIES WITH A CELL-FREE MICROFLUIDIC METHOD

Klaus Eyer,[1,3] Katharina Root,[1] Pascal E. Verboket,[1,2] Petra S. Dittrich[1,2]

[1]Department of Chemistry and Applied Biosciences, ETH Zurich (Switzerland)

[2]Department of Biosystems Science and Engineering, ETH Zurich (Switzerland)

[3]current addres: Laboratoire Colloïdes et Matériaux Divisés, ESPCI (France)

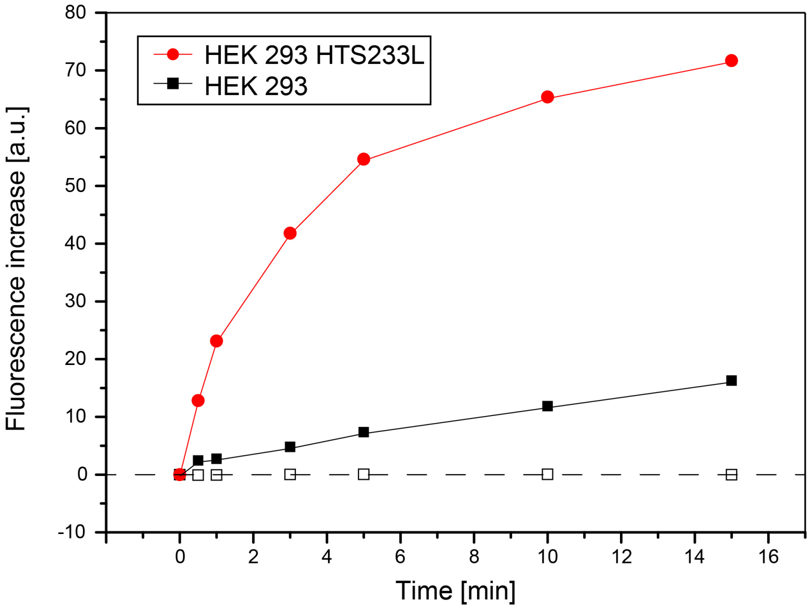

SI Fig. 1: Binding of labelled LH to vesicles derived from HEK293HTS233L and HEK293 cells. The graph shows the kinetic binding of labelled LH to LHHCG receptor positive (red circles) and negative (black squares) vesicles immobilized on-chip. In comparison, a channel without immobilized vesicles is shown (hollow squares). To obtain the curves, a concentration of LH of 2 nM and a flow rate of 10 nM was used.

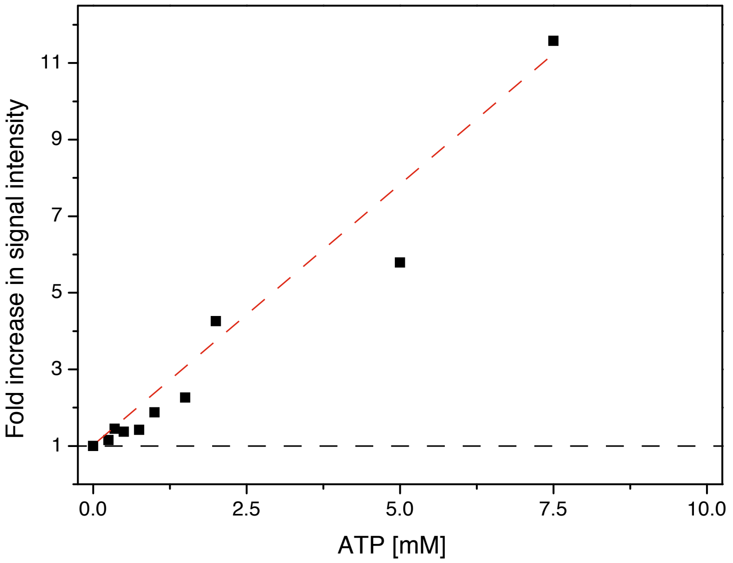

SI Fig. 2: Influence of externally added ATP on the signal. As shown here, integrated signal intensities are increasing with the amount of externally added ATP to the test solutions (n=1). For more explanation, please refer to the text. The dashed line does not represent a fit line, rather showing the direction in which the system is developing at higher concentrations.

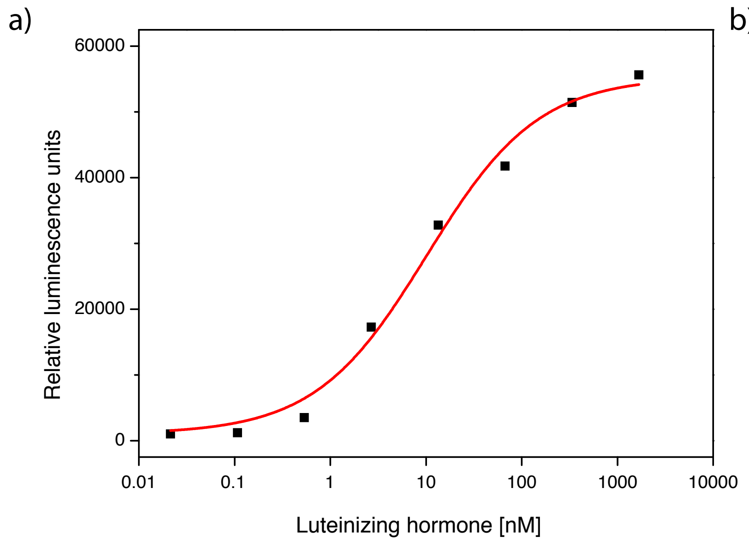

SI Fig. 3: Results from cell stimulation. a) Cells are responding to a stimulus with luteinizing hormone with the production of bioluminescence in a dose-response like behavior (n=2). b) Leaving the LH concentration constant, but adding an increasing amount of LH antibody, the luminescence decreases. Therefore, the antibody is decreasing the concentration of free LH that is able to interact with the receptor; the antibody is neutralizing the effect of the hormone (n=2).

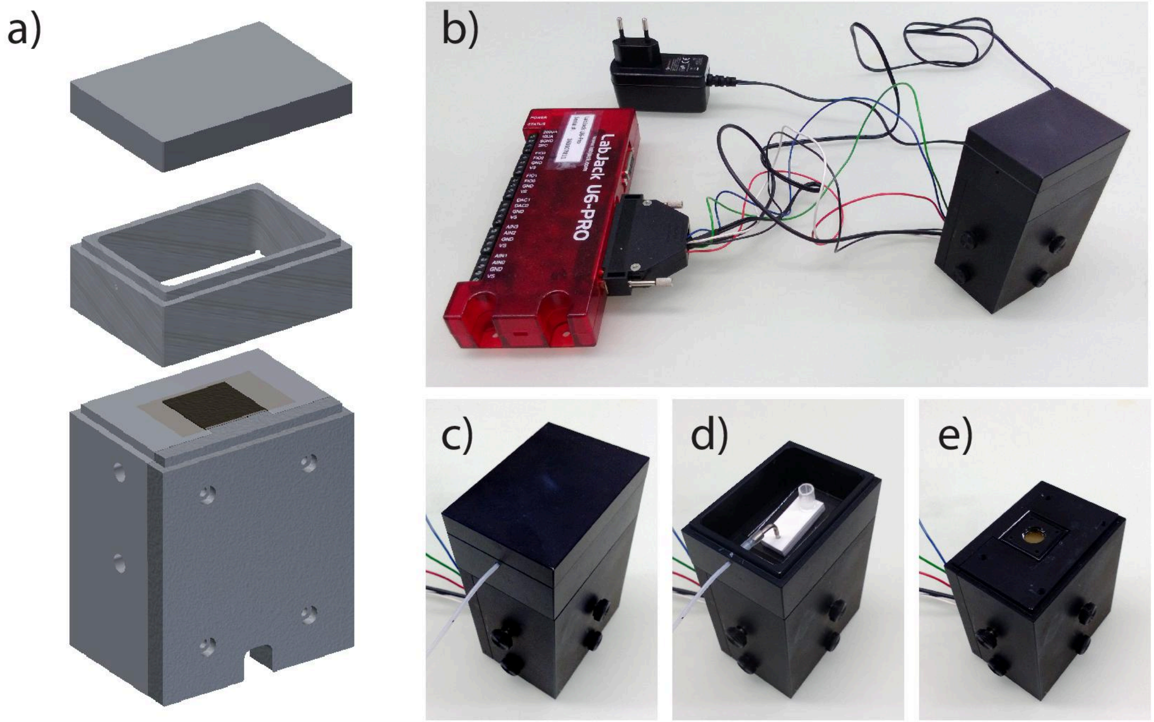

SI Fig. 4: Box for luminescence measurements. a) CAD drawings of the box. The box consists of three elements (top to bottom): a lid, a spacer with holes for fluid connections and a base element that harbours the PMT. b) Final setup. The picture shows the box connected to an analog to digital converter (shown in red) that is normally connected to a computer to read out the signal. c-e) The image series shows the closed box, without lid and without chip and spacer element. On e), the sensing are of the PMT is visible.

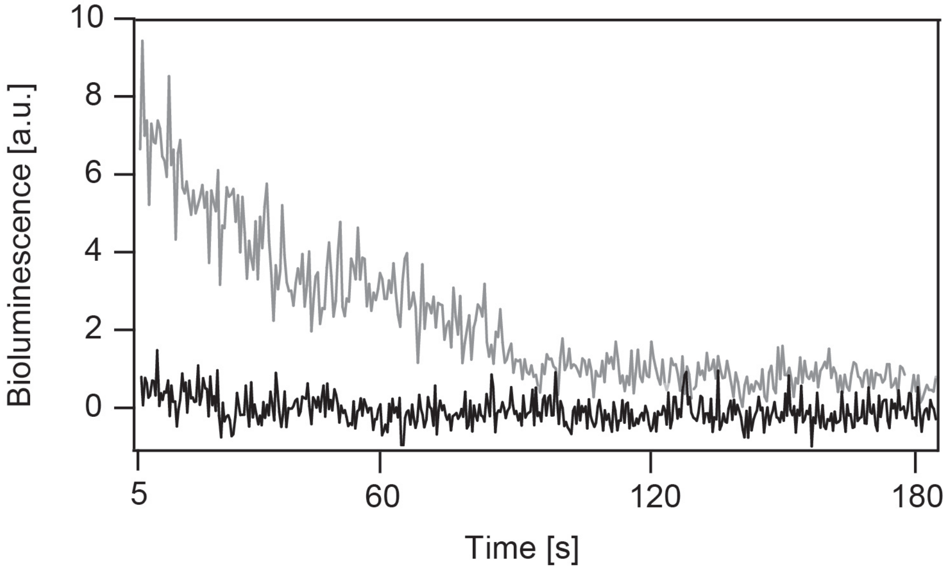

SI Fig. 5: Raw data traces from the bioluminescence measurement setup. The figure shows a raw data trace for LH containing sample (grey, 94 ng/ml), and the trace corresponding to 0 ng/ml LH (black). Interestingly, the curve is relatively similar to the one obtained using calcium measurements in Figure 2D due to the direct link of the calcium concentration on the activity of clytrin. LH was added at time zero, and measurements were obtained from second 5 onwards for 3 minutes. The lag-phase is due to the opening and closing of the box for the addition of the solution to the reservoir.

SI Table 1: Dynamic light scattering data of produced vesicles, measured wtih a Zetasizer 3000, Malvern Instruments

Measured diameter nm	Percentage in class
< 400	0
400	0
500	0
600	25
700	50
800	25
900	0
1000	0
> 1000	< 1

Characterization of the HTS233L LHHCG cell line and the antibody

In first experiments, the cells were tested for their luminescence response to LH, and the purchased antibody for its neutralizing ability thereof. Furthermore, the generated cellular dose-response curve served as a standard curve for comparison with vesicle experiments later on. Here, cells were seeded into µslides (ibidi, Germany) and loaded with colenterazine hcp in HBSS as described in the cell line manual. After the incubation time, luteinizing hormone was added to the cells and the generated luminescence was recorded. Indeed, the cells are responding to the stimulus with the production of bioluminescence with an EC_{50} value of 10.2 ± 2.4 nM (SI Fig. 1 a). As expected, the experiment shows a high cellular sensitivity to the presence of LH. Next, the supplied solution was spiked with different concentrations of the purchased anti-LH antibody. As visible in SI Fig. 1 b, the recorded luminescence signal decreased with increasing concentration of antibody, indicating that the antibody is able to reduce the concentration of LH that is able to interact with the receptor, therefore neutralizing the hormones' effect.

Calculations for clinical relevant samples

However, the actual LOD of the nAb present in clinical samples will not only depend on its concentration, but also on its dissociation constant. The commercial antibody used in this study has a fairly high affinity to LH (K_d 1.5 nM). In contrast, the dissociation constant of endogenously produced antibodies can vary from high pM to low µM values, and the concentration in serum during an active immune response may vary from low to high nM concentrations. For example, in a clinical report about neutralizing antibodies against HCG [35], the clinicians found a low affinity antibody (K_d 714 nM), but the antibody was present in a rather high concentration of 418 nmol/L. Based on the results with our method, we can calculate the limit of detection for antibodies with higher dissociation constants (Fig. 5).

We calculated the limit of detection of the method for different dissociation constants of the antibody using the following relation:

$$K_d = \frac{[LH]_{eq}([nAb]_{sample} - [nAb]_{bound})}{[nAb]_{bound}} \Rightarrow [nAb]_{sample} = \frac{[nAb]_{bound}}{[LH]_{eq}} K_d + [nAb]_{bound} \qquad \text{(SI 1)}$$

with $[LH]_{eq}$ as the free concentration of LH at equilibrium, $[nAB]_{sample}$ as the total antibody concentration, $[nAB]_{bound}$ as the bound antibody concentration. Knowing the K_d of our used antibody (1.53 nM) and the total antibody concentration (0.44 nM) as well as the total concentration of LH (1.785 nM), we can calculate using a free concentration of LH of 1.563 nM, i.e. 0.222 nM are bound. This free concentration is then used for the calculation of total antibody concentrations at different K_d values, i.e. $[nAB]_{bound}$ = 0.222 nM and $[LH]_{eq}$ = 1.563 nM.

When compared to the example mentioned above, the method would be sufficiently sensitive for the detection of the nAb. This finding makes us confident that the analytical setup is sensitive enough for the analysis of clinical samples, the next step in that has to be done in order to show the usability of the platform.
